# The Pathogen-Host Interactions database (PHI-base): additions and future developments

**DOI:** 10.1093/nar/gku1165

**Published:** 2014-11-20

**Authors:** Martin Urban, Rashmi Pant, Arathi Raghunath, Alistair G. Irvine, Helder Pedro, Kim E. Hammond-Kosack

**Affiliations:** 1 Department of Plant Biology and Crop Science, Rothamsted Research, Harpenden, Herts, AL5 2JQ, UK; 2 Molecular Connections Private Limited, Basavanagudi, Bangalore 560 004, Karnataka, India; 3 Department of Computational and Systems Biology, Rothamsted Research, Harpenden, Herts, AL5 2JQ, UK; 4 European Bioinformatics Institute, European Molecular Biology Laboratory, Wellcome Trust Genome Campus, Hinxton, Cambridge, CB10 1SD, UK

## Abstract

Rapidly evolving pathogens cause a diverse array of diseases and epidemics that threaten crop yield, food security as well as human, animal and ecosystem health. To combat infection greater comparative knowledge is required on the pathogenic process in multiple species. The Pathogen-Host Interactions database (PHI-base) catalogues experimentally verified pathogenicity, virulence and effector genes from bacterial, fungal and protist pathogens. Mutant phenotypes are associated with gene information. The included pathogens infect a wide range of hosts including humans, animals, plants, insects, fish and other fungi. The current version, PHI-base 3.6, available at http://www.phi-base.org, stores information on 2875 genes, 4102 interactions, 110 host species, 160 pathogenic species (103 plant, 3 fungal and 54 animal infecting species) and 181 diseases drawn from 1243 references. Phenotypic and gene function information has been obtained by manual curation of the peer-reviewed literature. A controlled vocabulary consisting of nine high-level phenotype terms permits comparisons and data analysis across the taxonomic space. PHI-base phenotypes were mapped via their associated gene information to reference genomes available in Ensembl Genomes. Virulence genes and hotspots can be visualized directly in genome browsers. Future plans for PHI-base include development of tools facilitating community-led curation and inclusion of the corresponding host target(s).

## INTRODUCTION

**Figure 1.**
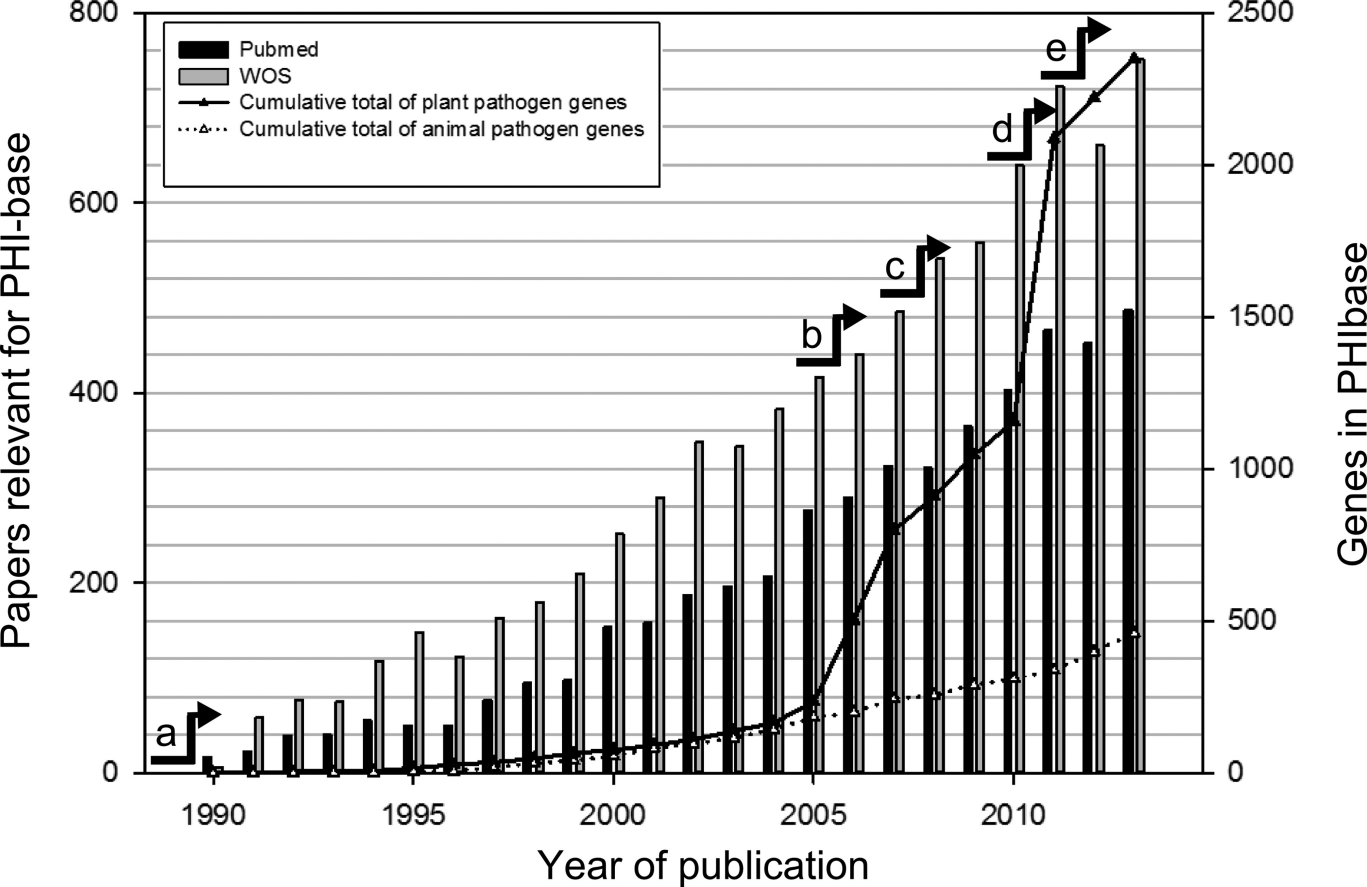
Growth of the number of published articles screened by keyword search for PHI-base and the number of phenotypically curated genes. This figure was generated from literature records retrieved at PubMed and Web of Science using the search terms ‘(fung*or yeast) and (gene or factor) and (pathogenicity or virulen* or avirulence gene*)’. Key events in the history of functional gene analysis of pathogen-host interactions include: a, identification of the first avirulence gene (4); b, >1500 genome sequencing projects listed in the GOLD database (5); c, genome-wide functional analysis of pathogenicity genes in the rice blast fungus *Magnaporthe oryzae*; d, the first host-induced gene silencing (HIGS) study involving an obligate biotrophic species (6); e, genome-wide functional analysis of all transcription factors and protein kinases predicted in the cereal infecting fungus *Fusarium graminearum* (7,8).

Existing and emerging infectious diseases are a major concern to plant, animal and human health, threaten global food security and increasingly affect the biodiversity of natural ecosystems (1,2). Although the diseased state is rare, myriads of micro-organisms and invertebrate pests have evolved the ability to infect another species, gain sufficient sustenance to colonize their chosen host(s) and then to reproduce and disseminate efficiently to reinitiate the infection process. In most host-pathogen, host-pest and host-parasite encounters, the host survives and the disease symptoms are limited to specific cell layers, tissues or organs. Only a few pathogenic species routinely kill their selected host(s). With the advent of molecular cloning methods 30 years ago, the functional analysis of genes in host-pathogen interactions became feasible. The aim of many of these studies is to identify the molecules and mechanisms involved in the disease formation process in an effort to develop remedial strategies to increase agricultural crop yield, to improve animal or human health or to maintain biodiversity within natural ecosystems. Since the publication of the first functional gene analyses in the early 1980s, which included the molecular characterization of the *avrA* avirulence gene from the bacterial pathogen *Pseudomonas syringae pv. glycinea* (PHI-base accession PHI:963) (3,4), many more genes involved in pathogen-host interactions have been identified and the number of publications has steadily increased (Figure 1). Further key events in the history of functional gene analysis of pathogen-host interactions include: in 2005, the listing of >1500 active genome sequencing projects by the Genomes Online Database (GOLD) (5); in 2007, the report of a genome-wide functional analysis study of pathogenicity genes in the rice blast fungus *Magnaporthe grisea*; in 2010, publication of the first host-induced gene silencing (HIGS) study involving an obligate biotrophic species (6); in 2011, the genome-wide functional analysis of all transcription factors and protein kinases in the cereal infecting fungus *Fusarium graminearum*(7,^8^).

Established in 2005, the pathogen-host interactions database (PHI-base) contains expertly curated molecular and biological information on genes proven to affect the outcome of pathogen-host interactions. Phenotypes can be assigned to the outcome of such interactions. Within PHI-base, genes are catalogued when their function in the pathogenic process has been tested through gene disruption and/or transcript level alteration experiments. These genes are termed pathogenicity genes if the effect on the phenotype is qualitative (disease/no disease). They are called virulence/aggressiveness genes if the effect is quantitative. Another category of genes increasingly catalogued in PHI-base are effector genes formerly known as avirulence genes. Effector genes either activate or suppress plant defence responses.

There are five key motivations to improve the data content of PHI-base and its taxonomic coverage: (i) In the post-genomics era and with the ever cheaper cost of whole-genome sequencing there is intense interest in comparative pathogen genomics to identify functionally homologous genes, as well as species-unique genes. (ii) The breadth and efficiency of both forward and reverse genetics analysis in plant- and animal-infecting pathogenic species has accelerated the pace of discovery, with generated mutants subject to intense investigation and scrutiny. In many interaction studies, model host species are used increasingly to save costs, but which may or may not yield results equivalent to those obtained in the natural host species. Thus, comparisons with known interactions in the natural host can be informative. (iii) Many gene sequences linked to a pathogenic process lack sufficient formal descriptive annotation, such as that provided by Gene Ontology (GO) (9). PHI-base provides a repository for such gene annotation. (iv) Increased species coverage across a wider taxonomic range permits the PHI-base data to be used *in silico* to predict with a higher level of confidence the repertoire of virulence associated genes in more species. (v) Finally, and most importantly, researchers require free and easy access to different types of interaction information to facilitate hypothesis generation and knowledge discovery.

Here, we report on a major increase in PHI-base gene content, new database features, integration with complementary databases and use cases. The original release of PHI-base was published in the NAR database issue in 2006 (10). A second NAR article in 2008 reviewed additional data and new features available within PHI-base version 3.0 (11). Since then usage of PHI-base has grown and the PHI-base website receives about 1500 hits per quarter, excluding internal users, with users located in ∼89 countries. Several other databases provide information which partially overlap with either the species data or biological information provided within PHI-base. These resources include the Fungal Virulence Factor Database (DFVF) (12), the e-Fungi project (13), Ensembl Genomes (14), the Oomycetes Transcriptomics Database (15), the Eukaryotic Pathogen Database Resources (EuPathDB) (16), FungiDB (17), the Host-Pathogen Interaction database on human viruses (HPIDB) (18), JGI-MycoCosm (19), PHIDIAS (20), PLEXdb (21) and the database on virulence factors of pathogenic bacteria (VFDB) (22). These complementary resources and their specialisms are summarized in Table 1. When used collectively, these databases provide prospective and existing users of PHI-base with a substantially enriched environment to pursue a wide range of simple to advanced *in silico* analyses on pathogenic organisms and the underlying pathogenic processes.

**Table 1.** Multispecies databases and websites involving plant, human and/or animal infecting pathogens which contain information complementary to the data in PHI-base

Name and ref^a^	URL (http://)	Comments
Broad-Fungal Genome Initiative	www.broadinstitute.org/scientific-community/science/projects/fungal-genome-initiative	Genome browsing and comparative analysis for several plant pathogen division
DFVF (12)	sysbio.unl.edu/DFVF	Fungal virulence factor database generated using text-mining of the PubMed database and Internet
e-Fungi (13)	www.cs.man.ac.uk/∼cornell/eFungi	Rich source of ESTs obtained by Sanger sequencing
Ensembl Genomes (14)	www.ensemblgenomes.org	Non-vertebrate species genomes portal with links to bacteria, fungi, metazoa, plants and protists
Ensembl Bacteria	bacteria.ensembl.org	Genomes of bacterial and archea
Ensembl Fungi	fungi.ensembl.org	Genomes of fungal species including fungal pathogens
Ensembl Protists	protists.ensembl.org	Genomes of protist species including Phytophthora
Oomycetes Transcriptomics Database (15)	www.eumicrobedb.org/transcripts	Oomycete genomes and transcriptomics
EuPathDB (16)	eupathdb.org	Human pathogens
FRAC	www.frac.info	All known chemical target sites used commercially for the control of pathogens
FungiDB (17)	fungidb.org	Fungal genomics database providing graphical tools for data mining
HPIDB (18)	agbase.msstate.edu	Fifteen human virus pathogens–protein-protein interaction data
JGI-MycoCosm (19)	genome.jgi.doe.gov/programs/fungi	A genome portal for 100s of pathogenic and non-pathogenic fungal species
Pathogen Portal	www.pathogenportal.org	Emerging or re-emerging pathogens, potential biowarfare or bioterrorism pathogens
PHIDIAS (20)	www.phidias.us	Medical fungal and bacterial pathogens
PhytoPath	www.phytopathdb.org	PhytoPath–32 Fungi, 14 Protists, 12 bacterial species linked to PHI-base
PLEXdb (21)	www.plexdb.org	Transcriptomics data only on plants, pathogens and during interactions
USDA	nt.ars-grin.gov/fungaldatabases	Description of all the known hosts of fungi which infect plants
VFDB (22)	www.mgc.ac.cn/VFs	Virulence factors of human and animal bacterial pathogens

^a^Reference provided where available.

## NEW FEATURES

### An expanded taxonomic range and controlled vocabulary

Version 3.0 released in 2007 contained information on bacterial, fungal and oomycete pathogens, as well as plant endophytes. Version 3.6 now also includes pathogenic plant infecting nematode and aphid pests and animal/human infecting parasites (Table 2). Between these versions of PHI-base, the total number of pathogenic species has risen from 95 to 160. The number of bacterial pathogens tripled over the same period. In addition, the number of obligate biotrophic species has increased from three to seven. To help PHI-base users become rapidly familiar with the biology of the wider range of pathogens and pests available, a full list of the pathogenic species covered in PHI-base version 3.6 is provided in Supplementary Table S1 along with their NCBI taxon identifier and both the natural and experimental host(s). The number of documented host species naturally infected by each pathogen and the identity of obligate biotrophs among the species is also described. This level of detail is provided to assist users in the selection of pathogenic species to include in comparative genomic analysis. An up-to-date version of Supplementary Table S1 is maintained on the PHI-base ‘About’ website, reflecting the data for each new release.

**Table 2.** Interactions in PHI-base version 3.6 grouped by either host species or pathogen species

Host/Entry type	Interactions
TOTAL^a^	4102
PROKARYOTES (55)^b^	804
Animal hosts (16)^c^	249 (31%)
*Salmonella spp.(3)^d^*	115
Plant hosts (29)	555 (69%)
*Xanthomonas spp. (10)*	300
*Pseudomonas spp.(7)*	161
*Erwinia amylovora*	29
*Plectobacterium spp. (3)*	10
EUKARYOTES (105)	3298
Animal hosts (20)	549 (16.6%)
*Ascomycetes* (17)	375
*Candida spp. (5)*	238
*Aspergillus fumigatus*	98
*Basidiomycetes* (4)	144
*Cryptococcus neoformans*	136
*Parasitic species* (5)^e^	30
Plant hosts (93)	2744 (83.2%)
*Ascomycetes* (60)	2384
*Fusaria* - cereal infecting *(7)*	1053
*Fusarium graminearum*	1042
*Magnaporthe spp.(3)*	575
*Botrytis spp.(2)*	205
*Fusaria* - dicot infecting *(6)*	93
*Cochliobolus (5)*	88
*Alternaria spp. (4)*	78
*Colletotrichium (9)*	48
*Stagnosporum nodorum*	44
*Zymoseptoria tritici*	42
*Basidiomycetes* (4)	261
*Ustilago maydis*	243
*Melampsori lini*	7
*Oomycetes* (8)	86
*Phytophthora spp. (5)*	53
*Hyaloperonospora spp.(2)*	30
Others (4)	13
*Aphids (2)*	10
*Nematodes (2)*	3
Fungal hosts (3)	4
Endophyte (1)	5
*Epichloe festucae*	5

^a^Only highly represented taxon groups are listed. For a complete list of species in the database see Supplementary Table S1.

^b^The table is divided into prokaryote and eukaryote host species. The species count number is listed in brackets.

^c^Host species are further divided into animal and plant host.

^d^Left-indented genera and species infect or belong to taxonomic group listed non-i**n**dented above. Only main representatives organisms are listed.

^e^Parasitic species are *Leishmania infantum, L. mexicana*, *Toxoplasma gondii*, *Trypanosoma brucei* and *T. cruzi.*

A new addition requested by users is the consistent use of a controlled vocabulary of high-level phenotyping terms (Table 3). Currently, nine phenotyping terms are used to permit consistent data retrieval, comparative phenomics across a wide taxonomic range and statistical analysis. Only one term is assigned per host-pathogen interaction. An interaction is defined as the function of one gene, on one host and one tissue type. The PHI-base phenotype terms selected are routinely used in research articles but mapping to GO terms is not supported due to their high-level nature. Since 2008, several new techniques for investigating gene product function have become more widely adopted. For example, for some obligate plant infecting pathogens, including *Blumeria* and *Puccinia* species which infect specific cereal hosts, a novel technique called host-induced gene silencing (HIGS) is used. In HIGS, an antisense construct is expressed from the host species and used to transiently silence a specific pathogen gene during the infection process, which if successful, results in an altered phenotypic outcome (23). The eight entries PHI:2896 to PHI:2903 were obtained for the *Blumeria graminis* f. sp. *hordei*–barley interaction using the new HIGS technique.

**Table 3.** Definitions for the nine high-level phenotype outcomes used in PHI-base

High-level phenotype outcome^a^	Definition
Loss of pathogenicity	The transgenic strain fails to cause disease that is observed in the wild type (i.e. qualitative effect).
Reduced virulence	The transgenic strain still causes some disease formation but fewer symptoms than the wild-type strain (i.e. a quantitative effect). Synonymous with the term reduced aggressiveness.
Unaffected pathogenicity	The transgenic strain which expresses altered levels of a specific gene product(s) causes the same level of disease compared to the wild-type reference strain.
Increased virulence (Hypervirulence)	The transgenic strain causes greater incidence or severity of disease than the wild-type strain.
Effector (plant avirulence determinant)	Some effector genes are required to cause disease on susceptible hosts but most are not. A plant pathogen-specific term which was previously referred to as a corresponding avirulence *(Avr)* gene. An effector gene is formally identified because its presence leads to the direct or indirect recognition of a pathogen in resistant host genotypes which possess the corresponding disease resistance *(R)* gene. Positive recognition leads to activation of plant defense and the pathogen either fails to cause disease or causes less disease. In the absence of the pathogen, effector delivery into a healthy plant possessing the corresponding *R* gene activates plant defense responses.
Lethal	The transgenic strain is not viable. The gene product is essential for life of the organism.
Enhanced antagonism	The transgenic strain shows greater endophytic biomass in the host and/or the formation of visible disease symptoms.
Resistant to chemical	The transgenic strain^b^ grows and/or develops normally when exposed to chemistry concentrations that are detrimental to the wild-type strain.
Sensitive to chemical	The transgenic strain which expresses either no or reduced levels of a specific gene product(s) or possesses a specific gene mutation(s), has the same ability^c^ as the wild-type strain to grow and develop when exposed to detrimental chemistry concentrations.

^a^Compared to wild-type reference strain (i.e. a direct isogenic strain comparison).

^b^Molecular studies on natural field isolate population are also considered, once the natural target site has been identified.

^c^On rare occasions increased sensitivity to chemistry has been observed.

### Additional content and species coverage

PHI-base version 3.6 contains information on 2875 genes, 4102 interactions, 110 host species and 160 pathogenic species. The pathogen species include 103 plant, 3 fungal and 54 animal infecting species. The organisms in the database cause 181 different diseases and were obtained from 1243 peer-reviewed references. The functional gene information included was curated from studies published between 1987 to the end of 2013. Details of the host and pathogen species coverage is given in Table 2 and Supplementary Table S1. One-third of the prokaryote interactions now involve a human pathogen, with the highest number of 115 interactions from *Salmonella* species. For plant infecting bacteria the highest numbers are 300 and 161 interactions from *Xanthomonas* and *Pseudomonas* species, respectively. The fungal pathogen interactions are dominated by the Ascomycetes (67 species) followed by the Basidiomycetes (8 species), providing 2759 and 405 interactions, respectively. The fungal interactions are also predominantly from plant infecting species (2645 interactions) compared to animal/human infecting species (519 interactions). The number of interactions from the eight oomycete species is far lower at 86, which are all from plant infecting species. The newly curated plant infecting nematodes and aphids and animal/human infecting parasites provide 43 interactions from 9 species. The new data is summarized by host type and pathogen species taxonomy in Table 2. The plant pathogen species providing the greatest number of interactions are the cereal infecting fungi *Fusarium graminearum*, *Magnaporthe oryzae* and *Ustilago maydis*, *Xanthomonas* bacteria and the dicotyledonous infecting fungus *Botrytis cinerea* and *Pseudomonas* bacteria. For animal/human infecting species the greatest number of interactions are provided by the fungi *Candida albicans* and *Cryptococcus neoformans* and the bacterium *Salmonella entrica* (Table 2).

The nine new high-level phenotypic outcome terms are defined in Table 3. These have been included in the advanced search to permit researchers to explore the database across a wide range of taxonomically diverse species which exhibit very varied pathogenic lifestyles. Only the entry types ‘effector’ and ‘enhanced antagonism’ are limited to plant infecting species. In total, 84 interactions from a total of 23 species have the outcome ‘increased virulence (hypervirulence)’. This expanding number is noteworthy and suggests that negative regulation of key pathogenicity processes commonly occurs during the infection and colonization of both plant and animal hosts. Also of interest are the 1224 interactions (29.8% of the entire database content) with the outcome ‘unaffected pathogenicity’. The majority of these have been reported for plant pathogens. These negative outcomes are usually presumed by the authors to indicate the gene product does not have a role in the pathogenic process under investigation or has arisen due to genetic redundancy, i.e. the function of a highly homologous gene replaces the function of the missing gene product under experimental evaluation. In some studies, the inclusion of double-gene deletion results has been able to clarify the situation. For example, the *Candida albicans* gene *PDE1* (PHI:857) has been implicated in virulence. The *PDE1* mutant alone is unaffected in pathogenicity. However, the double-gene deletion of *PDE1* and *PDE2* shows a more severe effect than deletion of the *PDE2* (PHI:856) gene on its own (24). In *Magnaporthe oryzae* (formerly called *M. grisea*) deletion of the individual genes *MoRgs1* (PHI:2192) and *MoRgs4* (PHI:2195) led to a reduced-virulence phenotype, but the double-gene deletion *rgs1 rgs4* mutant has a more severe ‘loss of pathogenicity’ phenotype (25). In the animal pathogen *Vibrio cholerae*, the effect of a triple mutation on biofilm formation and virulence was used to test the combined function of *tatA* (PHI:2415), *tatB* (PHI:2416), *tatC* (PHI:2417) and revealed this small gene family was required for virulence in mice (25). Going forward, the use of the ‘unaffected pathogenicity’ category in comparative species analyses will be particularly informative when the genes involved are present in only one copy per species. This approach will reveal which genes function in a species-specific or taxon clade-specific manner.

The high-level phenotypic outcomes for all interactions are summarized in Table 4. A total of 120 PHI-base accessions have been assigned the high-level phenotypic outcome ‘Essential (lethal)’. In these studies, mainly two types of experimental data were reported. First, in *Aspergillus fumigatus* a promoter replacement strategy was employed to construct conditional mutants. For these mutants the addition of ammonium into the nitrogen source switches off gene expression and this allows functional gene tests of essential genes (26). Secondly, in genome-wide gene replacement studies in *Gibberella zeae* no transformants were recovered in repeated experiments, while transformants were recovered for many other genes. Thus, authors considered that the gene's function was ‘essential for life’ (7,8).

**Table 4.** Number of interactions per phenotypic group in animal and plant hosts

Entry type	Animal host^a^	Plant host
Loss of pathogenicity	73	404
Reduced virulence^b^	542	1056
Increased virulence	33	51
Essential (lethal)	46	74
Unaffected pathogenicity^c^	80	1144
Effector	0	533
Enhanced antagonism	0	4
Resistance to chemistry	5	30
Sensitive to chemistry^d^	1	7

^a^Animal and plant-attacking pathogens are listed with their taxonomy ID and lifestyle in Supplementary Table S1.

^b^The three missing entries in this category have other host types.

^c^One entry in this category has a fungal host

^d^One entry in this category has a fish host.

A ‘mixed outcome’ of phenotypes can be assigned when the transgenic mutants generated are tested on either multiple host species or different tissues/organs of the same host species. Different outcomes on hosts belonging to different kingdoms potentially indicate a differential host requirement. For example, *Fusarium oxysporum* is able to systemically infect tomato plants and immune-compromised mice. The PHI-base entries PHI:215, PHI-285 and PHI:315 reveal a differential requirement for cell-signalling and cell wall formation of three genes during the pathogenesis of plant and animal hosts.

### Integration with other database sources

PHI-base is a gene-centric database. Each gene has its own PHI-base accession number. One advantage of this design is that phenotypic information is directly linked to a specific gene. This phenotypic information can then easily be mapped to genomes. Additional information, such as GO terms and protein structure information, is then extracted from other databases. In our current curation we prioritise the use on UniProt accessions (27) to facilitate subsequent bioinformatics analysis. During the curation process, our biocurators map reported EMBL or GenBank accessions to existing UniProt identifiers, where these exist. However, for species where protein accessions are not available in UniProt at the time of curation and authors did not provide GenBank accession numbers in their studies only limited or no information on the gene/protein can be provided in PHI-base until this information becomes available.

Whole-genome information is increasingly available for plant and animal pathogens. We have mapped phenotypes in PHI-base via their gene accessions to reference genomic sequences available in Ensembl Genomes sites for fungi, protist (including oomycetes) and bacteria (26). In total, 1550 out of 2047 interactions involved in plant pathogenesis from pathogens with an available reference genome have been mapped to Ensembl Genomes. The remainder of the PHI-base accessions are either associated with only genetic data or the genome sequence information is still missing, or are associated with previously reported sequences and isolates that differ from those in the published reference genomes. Work is continuing to resolve these cases.

### Functional analysis of PHI-base accessions

**Figure 2.**
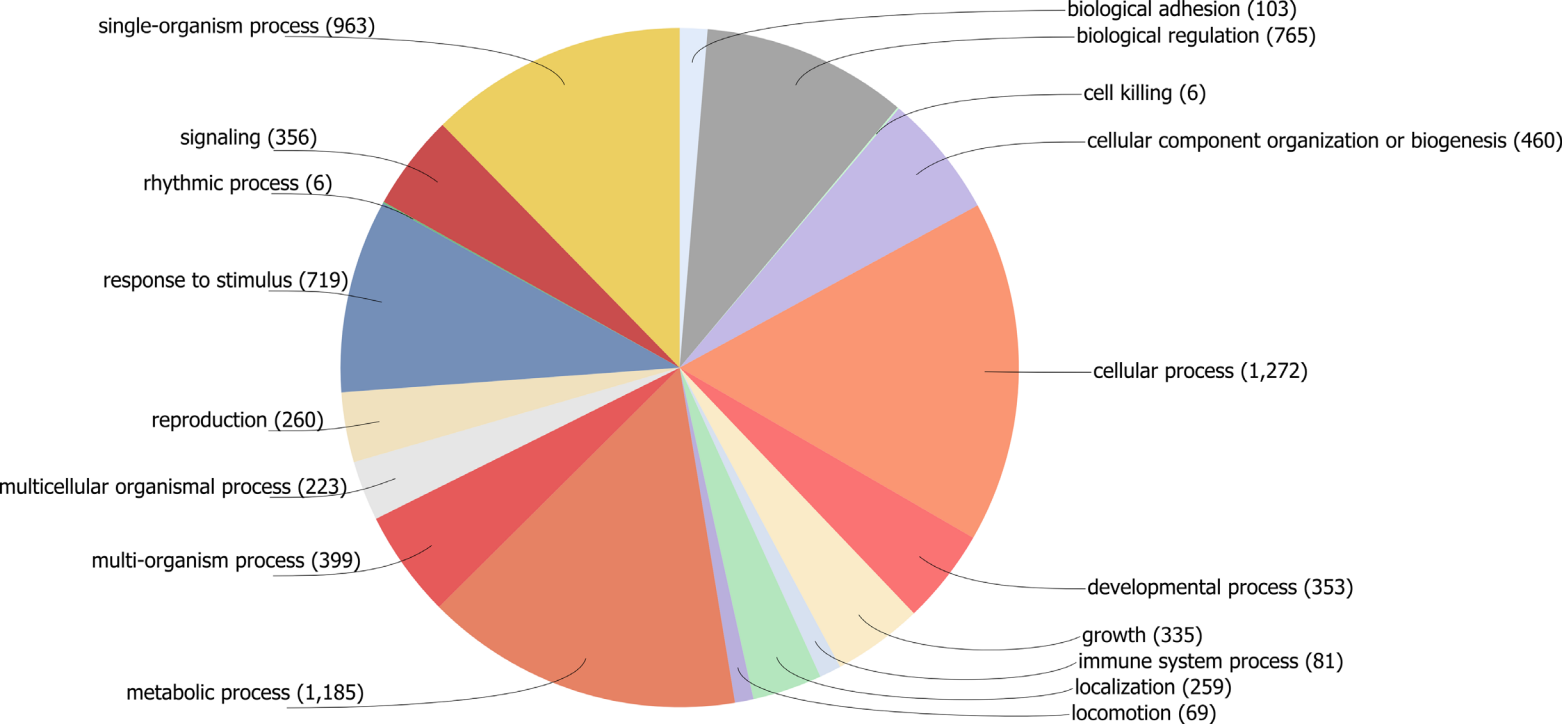
GO terms assigned to PHI-base accessions in Version 3.6 mapped to a biological process.

The entire contents of PHI-base are available to users from the ‘Download’ section, where sequence information is available for 2527 PHI-base accessions. We surveyed the content of PHI-base accessions by cataloguing the protein accessions using their GO classification using Blast2GO software and standard parameters (28). GO terms were assigned to 63% of PHI-base accessions (Figure 2). For a total of 37% (929 proteins), no GO annotation could be made. Many of these accessions are species-specific proteins and are effectors. The major GO categories assigned included (i) metabolic processes, (ii) cellular processes, such as cell communication, and (iii) single-organism processes, such as cell proliferation, filamentous growth and pigmentation. Microbial pigments in pathogens are known to provide protection against ultraviolet radiation, host-defence products and other stresses encountered during host invasion.

The category ‘cell killing’ was only assigned to six accessions and included Pseudomonas effectors and the *Vibrio cholerae* enterotoxin. This low number is an unexpected result because for many of the host-pathogen interactions catalogued in PHI-base at some point host cell death occurs, i.e. in interactions involving pathogens with a necrotrophic or hemibiotrophic lifestyle.

## TECHNICAL DEVELOPMENTS, CURATION AND OUTREACH

### Data curation and release management

In the NAR 2008 article (11) we provided the details of the curation procedure in use. This procedure is still in place. However, due to the increasing volume of literature requiring curation (Figure 1) we now use additional procedures. Primarily, papers are found in the literature databases Web of Science and PubMed using the keyword search terms: (fung*or yeast) and (gene or factor) and (pathogenicity or virulen* or avirulence gene*) (29). Text mining is not employed due to the fact that relevant information has to be extracted by analysing figures, tables and text in the peer-reviewed articles. This task can only be done by trained biocurators with a strong understanding of the research area. PHI-base relies heavily on support of the scientific community to suggest relevant articles for curation and for the subsequent quality control of entries. The PHI-base team does not have any individual member solely dedicated to data curation. Instead, team members curate data on a part-time basis and when the need arises. In an effort to close a curation gap for articles published between 1984 and 2014, a collaboration was established with the curation scientists at Molecular Connections, Bangalore, India. The biocurators give priority to author assigned gene function over computational transferred annotation, such as GO terms. The author-assigned function is frequently extracted from either title or abstract. Experts from the scientific community are invited on a regular basis to verify new records before uploading into the database and provide quality control.

### Mapping PHI-base phenotypes to Ensembl Genomes

**Figure 3.**
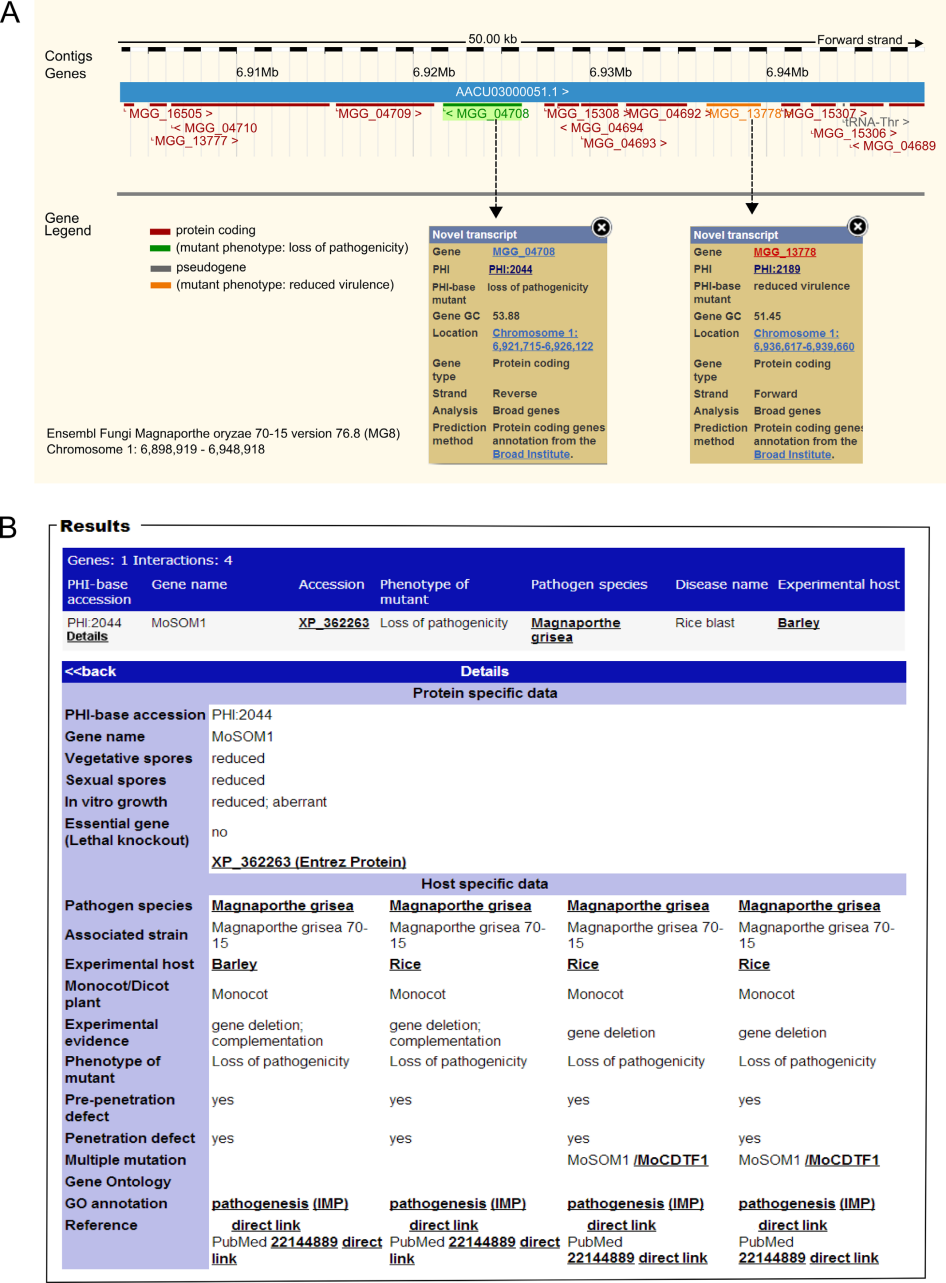
Inspection of gene function using the Ensembl genome browser. (A) Displayed is a small chromosomal region in *Magnaporthe oryzae* showing two genes involved in pathogenicity (as annotated in PHI-base) in their genomic context (viewable in the Ensembl browser, in the transcript display). A colour code indicates the annotated role of each gene, green ‘loss of pathogenicity’ and orange ‘reduced virulence’. (B) By selecting each colour-coded MGG transcript ID, information is revealed on the associated gene deletion study curated in the PHI-base database.

Through the cross-referencing with Ensembl Genomes (http://ensemblgenomes.org) PHI-base annotations can now be visualized directly in their genomic context, identifying features, such as pathogenicity islands through a simple system of colour coding using the nine high-level phenotyping terms. This new way to explore the data in PHI-base is shown in Figure 3. The phenotyping term ‘mixed outcome’ is also used to identify genes where a range of interaction outcomes have been identified depending on the host species and/or tissue type evaluated.

## APPLICATIONS OF PHI-BASE

PHI-base use has been cited in over 100 peer-reviewed publications. These publications are listed in year order in the ‘About’ section of the database. Recently published PHI-base use cases include genome mining and comparative genomics (30,^31^), the selection and functional testing of candidate virulence factors in newly sequenced fungal and nematode pathogens of agricultural importance (10,11) and studies investigating the subtle differences between pathogen and biocontrol species (23). In Table 5 the main uses of PHI-base are given along with literature examples (14,^30^–^46^). In the past 4 years we have observed a gradual shift in PHI-base use, with an increase in the number of larger comparative gene function studies and investigations reporting the *in silico* prediction of virulence-associated genes.

**Table 5.** PHI-base uses that have often appeared in the peer-reviewed literature

Use case	Type of research study	Example reference
1	Annotation and candidate gene selection	
	Large scale forward genetics screens	(32,33)
	Transcriptome studies (RNAseq, microarrays, ESTs)	(34,36,45)
	Full and partial genome annotation, genome mining	(30,31,37)
2	Predictive bioinformatics analyses: Networks, protein-protein interaction mapping	(35,38,39)
3	Complementary databases	(14,40,41)
4	Review articles	(42,44,46)
5	Single gene function studies	
	Inter-comparisonand inter-comparison of gene mutants within and between species	(43)

## FORTHCOMING DEVELOPMENTS

### Tools for community-led curation

A big challenge facing all biological databases is the growing quantity of data and the relative difficulty of obtaining resources to curate the knowledge that derives from it. For the pathogen-host interaction community the scale of the problem is considerable (Figure 1). One solution is to encourage community-based curation, particularly by the authors of scientific publications, who may be motivated to have their work correctly represented within the database, and who are the experts in their own specialist domains (although they may not be expert in the conventions in use within the database). Inclusion of studies in PHI-base also improves their visibility and accessibility. PHI-base has a curation model based on community contribution, although hitherto, this has involved certain collaborators curating many papers in their own area of expertise, after prior training in the data entry tools. A more scalable model would allow all users to directly curate their own papers without prior training. A new easy-to-use web-based interface for direct access by the wider community is currently in development.

The PHI-base web-based curation tool will facilitate curation of pathogen-host interactions from peer reviewed literature into PHI-base by the authors doing the experimental analyses. This curation tool will be based on the recently developed Canto tool, an online tool that supports functional gene annotation (47). Canto is part of the Generic Model Organism Database project, which provides a suite of open software for managing genetic data (http://www.gmod.org). Canto has proven effective for the community-based curation of data for the fission yeast database, PomBase (http://www.pombase.org) (48). The PHI-base curation tool will use ontological data from a variety of sources, most notably from the Open Biological and Biomedical Ontologies Foundry (http://www.obofoundry.org) (49). However, some terminology is specific to the nature of the interactions captured in PHI-base, so will require the development of new controlled vocabularies for this purpose. For example, an ‘interaction evidence’ ontology will be created to specify the evidence for pathogen-host interactions, thus complimenting the gene-centric data from the GO. Also, in addition to the controlled high level vocabulary above describing the phenotype of the pathogen (Table 4), a similar controlled vocabulary can be created to describe the affect the interaction has on the host organism. To ensure quality and consistency of the curated data, all annotations will still be approved by a curator or expert with knowledge of the species and the captured data.

### Tools for data mining

We are currently developing a new tool for the analysis and extraction of whole genomic data from plant pathogens as part of the PhytoPath project (http://www.phytopathdb.org) using the data warehousing framework BioMart (50), allowing users to mine genomic data (for sequence and annotation) across multiple species based on PHI-base annotations in conjunction with other annotations. The tool is expected to be launched before the end of 2015.

### Other activities

Our intention is to extend the taxonomic range available within PHI-base to ∼200 host-infecting species within the next 2 years. At this level of species coverage detailed analyses within and between specific groups of pathogens with different infection strategies, host ranges, taxonomic assignments or between pathogenic and closely related non-pathogenic, endophytic or symbiotic lifestyles should be feasible.

In the next phase of curation a greater emphasis will be placed on the effector literature which should increase the number of interactions from bacterial, oomycete and obligate biotrophic species. To accompany this development, the curation of the corresponding host target(s), i.e. initial molecular partner in the host has commenced and this important information should soon be available. For example, various bacterial effectors including AvrRpm1 (PHI:977) and AvrRpt2 (PHI:979) are delivered into the plant cytoplasm via the bacterial type III secretion system. These effectors interact with the Arabidopsis protein RIN4 (51). These protein interaction data sets are of growing importance in the analysis of host-pathogen, host-pest and host-parasite interactions as they typically represent communication events that have co-evolved between biological kingdoms. For example, Arabidopsis mutants harbouring T-DNA insertions within different host targets of specific effectors were found to exhibit an enhanced disease resistance (*edr*) phenotype to both powdery and downy mildews (52). In addition, by the inclusion of the corresponding host targets, more effectors from obligate biotrophic species can be curated into PHI-base. These effectors are rigorously tested for their role in pathogenicity using a range of other techniques, but not those involving the generation of stable pathogen transformants.

### Database access and feedback

PHI-base can be freely accessed at http://www.phi-base.org. The complete database can be downloaded from the ‘Download’ section. Prior to downloading the entire database to create local Basic Local Alignment Search Tool databases or for other bioinformatics applications (Table 5), users are asked to fill in a registration form. This allows PHI-base to monitor the number of academic and industrial users, a requirement by our sponsors.

User support can be obtained from this email: contact@phi-base.org. Please use this email address if you wish to provide new data for inclusion in PHI-base, if you are an expert willing to assist with curation, for the nomination of peer-reviewed papers to be curated, or if you can provide suggestions for improvement to the PHI-base website.

To increase the awareness of PHI-base developments within the community and for users to be notified when new releases occur, we have developed a PHI-base user mailing list (users@lists.phi-base.org). Users can subscribe from a link on the PHI-base website in the ‘Help’ section, or directly by going to https://www.lists.rothamsted.ac.uk/mailman/listinfo/users.

## SUPPLEMENTARY DATA

Supplementary Data are available at NAR Online.

## References

[1] Dangl J.L., Horvath D.M., Staskawicz B.J. (2013). Pivoting the plant immune system from dissection to deployment. Science.

[2] Fisher M.C., Henk D.A., Briggs C.J., Brownstein J.S., Madoff L.C., McCraw S.L., Gurr S.J. (2012). Emerging fungal threats to animal, plant and ecosystem health. Nature.

[3] Napoli C., Staskawicz B. (1987). Molecular characterization and nucleic acid sequence of an avirulence gene from race 6 of *Pseudomonas syringae pv. glycinea*. J. Bacteriol..

[4] Staskawicz B.J., Dahlbeck D., Keen N.T. (1984). Cloned avirulence gene of *Pseudomonas syringae pv. glycinea* determines race-specific incompatibility on *Glycine max (L.)*. Merr. Proc. Natl. Acad. Sci. U.S.A..

[5] Liolios K., Tavernarakis N., Hugenholtz P., Kyrpides N.C. (2006). The Genomes On Line Database (GOLD) v.2: a monitor of genome projects worldwide. Nucleic Acids Res..

[6] Nowara D., Gay A., Lacomme C., Shaw J., Ridout C., Douchkov D., Hensel G., Kumlehn J., Schweizer P. (2010). HIGS: host-induced gene silencing in the obligate biotrophic fungal pathogen Blumeria graminis. Plant cell.

[7] Son H., Seo Y.S., Min K., Park A.R., Lee J., Jin J.M., Lin Y., Cao P., Hong S.Y., Kim E.K. (2011). A phenome-based functional analysis of transcription factors in the cereal head blight fungus, *Fusarium graminearum*. PLoS Pathog..

[8] Wang C., Zhang S., Hou R., Zhao Z., Zheng Q., Xu Q., Zheng D., Wang G., Liu H., Gao X. (2011). Functional analysis of the kinome of the wheat scab fungus *Fusarium graminearum*. PLoS Pathog..

[9] Gene Ontology Consortium (2013). Gene Ontology annotations and resources. Nucleic Acids Res..

[10] Winnenburg R., Baldwin T.K., Urban M., Rawlings C., Kohler J., Hammond-Kosack K.E. (2006). PHI-base: a new database for pathogen host interactions. Nucleic Acids Res..

[11] Winnenburg R., Urban M., Beacham A., Baldwin T.K., Holland S., Lindeberg M., Hansen H., Rawlings C., Hammond-Kosack K.E., Kohler J. (2008). PHI-base update: additions to the pathogen host interaction database. Nucleic Acids Res..

[12] Lu T., Yao B., Zhang C. (2012). DFVF: database of fungal virulence factors. Database.

[13] Hedeler C., Wong H.M., Cornell M.J., Alam I., Soanes D.M., Rattray M., Hubbard S.J., Talbot N.J., Oliver S.G., Paton N.W. (2007). e-Fungi: a data resource for comparative analysis of fungal genomes. BMC Genom..

[14] Kersey P.J., Allen J.E., Christensen M., Davis P., Falin L.J., Grabmueller C., Hughes D.S., Humphrey J., Kerhornou A., Khobova J. (2014). Ensembl Genomes 2013: scaling up access to genome-wide data. Nucleic Acids Res..

[15] Tripathy S., Deo T., Tyler B.M. (2012). Oomycete Transcriptomics Database: a resource for oomycete transcriptomes. BMC Genom..

[16] Aurrecoechea C., Brestelli J., Brunk B.P., Fischer S., Gajria B., Gao X., Gingle A., Grant G., Harb O.S., Heiges M. (2010). EuPathDB: a portal to eukaryotic pathogen databases. Nucleic Acids Res..

[17] Stajich J.E., Harris T., Brunk B.P., Brestelli J., Fischer S., Harb O.S., Kissinger J.C., Li W., Nayak V., Pinney D.F. (2012). FungiDB: an integrated functional genomics database for fungi. Nucleic Acids Res..

[18] Kumar R., Nanduri B. (2010). HPIDB–a unified resource for host-pathogen interactions. BMC Bioinformatics.

[19] Grigoriev I.V., Nikitin R., Haridas S., Kuo A., Ohm R., Otillar R., Riley R., Salamov A., Zhao X., Korzeniewski F. (2014). MycoCosm portal: gearing up for 1000 fungal genomes. Nucleic Acids Res..

[20] Xiang Z., Tian Y., He Y. (2007). PHIDIAS: a pathogen-host interaction data integration and analysis system. Genome Biol..

[21] Dash S., Van Hemert J., Hong L., Wise R.P., Dickerson J.A. (2012). PLEXdb: gene expression resources for plants and plant pathogens. Nucleic Acids Res..

[22] Chen L., Xiong Z., Sun L., Yang J., Jin Q. (2012). VFDB 2012 update: toward the genetic diversity and molecular evolution of bacterial virulence factors. Nucleic Acids Res..

[23] Lee W.S., Hammond-Kosack K.E., Kanyuka K. (2012). Barley stripe mosaic virus-mediated tools for investigating gene function in cereal plants and their pathogens: virus-induced gene silencing, host-mediated gene silencing, and virus-mediated overexpression of heterologous protein. Plant Physiol..

[24] Wilson D., Tutulan-Cunita A., Jung W., Hauser N.C., Hernandez R., Williamson T., Piekarska K., Rupp S., Young T., Stateva L. (2007). Deletion of the high-affinity cAMP phosphodiesterase encoded by *PDE2* affects stress responses and virulence in *Candida albicans*. Mol. Microbiol..

[25] Zhang L., Zhu Z., Jing H., Zhang J., Xiong Y., Yan M., Gao S., Wu L.F., Xu J., Kan B. (2009). Pleiotropic effects of the twin-arginine translocation system on biofilm formation, colonization, and virulence in *Vibrio cholerae*. BMC Microbiol..

[26] Hu W., Sillaots S., Lemieux S., Davison J., Kauffman S., Breton A., Linteau A., Xin C., Bowman J., Becker J. (2007). Essential gene identification and drug target prioritization in *Aspergillus fumigatus*. PLoS Pathog..

[27] UniProt Consortium (2014). Activities at the Universal Protein Resource (UniProt). Nucleic Acids Res..

[28] Gotz S., Garcia-Gomez J.M., Terol J., Williams T.D., Nagaraj S.H., Nueda M.J., Robles M., Talon M., Dopazo J., Conesa A. (2008). High-throughput functional annotation and data mining with the Blast2GO suite. Nucleic Acids Res..

[29] Baldwin T.K., Winnenburg R., Urban M., Rawlings C., Koehler J., Hammond-Kosack K.E. (2006). The pathogen-host interactions database (PHI-base) provides insights into generic and novel themes of pathogenicity. MPMI.

[30] Hane J.K., Anderson J.P., Williams A.H., Sperschneider J., Singh K.B. (2014). Genome sequencing and comparative genomics of the broad host-range pathogen *Rhizoctonia solani* AG8. PLoS Genet..

[31] Danchin E.G., Arguel M.J., Campan-Fournier A., Perfus-Barbeoch L., Magliano M., Rosso M.N., Da Rocha M., Da Silva C., Nottet N., Labadie K. (2013). Identification of novel target genes for safer and more specific control of root-knot nematodes from a pan-genome mining. PLoS Pathog..

[32] Jeon J., Park S.Y., Chi M.H., Choi J., Park J., Rho H.S., Kim S., Goh J., Yoo S., Choi J. (2007). Genome-wide functional analysis of pathogenicity genes in the rice blast fungus. Nat. Genet..

[33] Cai Z., Li G., Lin C., Shi T., Zhai L., Chen Y., Huang G. (2013). Identifying pathogenicity genes in the rubber tree anthracnose fungus *Colletotrichum gloeosporioides* through random insertional mutagenesis. Microbiol. Res..

[34] Vargas W.A., Martin J.M.S., Rech G.E., Rivera L.P., Benito E.P., Diaz-Minguez J.M., Thon M.R., Sukno S.A. (2012). Plant defense mechanisms are activated during biotrophic and necrotrophic development of *Colletotricum graminicola* in maize. Plant Physiol..

[35] Sperschneider J., Gardiner D.M., Taylor J.M., Hane J.K., Singh K.B., Manners J.M. (2013). A comparative hidden Markov model analysis pipeline identifies proteins characteristic of cereal-infecting fungi. BMC Genom..

[36] Thakur K., Chawla V., Bhatti S., Swarnkar M.K., Kaur J., Shankar R., Jha G. (2013). De novo transcriptome sequencing and analysis for Venturia inaequalis, the devastating apple scab pathogen. Plos One.

[37] Lefebvre F., Joly D.L., Labbe C., Teichmann B., Linning R., Belzile F., Bakkeren G., Belanger R.R. (2013). The transition from a phytopathogenic smut ancestor to an anamorphic biocontrol agent deciphered by comparative whole-genome analysis. Plant Cell.

[38] Schleker S., Garcia-Garcia J., Klein-Seetharaman J., Oliva B. (2012). Prediction and comparison of Salmonella-human and Salmonella-Arabidopsis interactomes. Chem. Biodiv..

[39] Liu X., Tang W.H., Zhao X.M., Chen L. (2010). A network approach to predict pathogenic genes for *Fusarium graminearum*. PLoS One.

[40] Kour A., Greer K., Valent B., Orbach M.J., Soderlund C. (2012). MGOS: development of a community annotation database for *Magnaporthe oryzae*. MPMI.

[41] Bleves S., Dunger I., Walter M.C., Frangoulidis D., Kastenmuller G., Voulhoux R., Ruepp A. (2014). HoPaCI-DB: host-Pseudomonas and Coxiella interaction database. Nucleic Acids Res..

[42] Van De Wouw A.P., Howlett B.J. (2011). Fungal pathogenicity genes in the age of ‘omics’. Mol. Plant Pathol..

[43] Doehlemann G., Reissmann S., Assmann D., Fleckenstein M., Kahmann R. (2011). Two linked genes encoding a secreted effector and a membrane protein are essential for *Ustilago maydis*-induced tumour formation. Mol. Microbiol..

[44] Dickman M.B. (2007). Subversion or coersion? Pathogenic deteminants in fungal phytopathogens. Fungal Biol. Rev..

[45] Zhang Y., Zhang K., Fang A., Han Y., Yang J., Xue M., Bao J., Hu D., Zhou B., Sun X. (2014). Specific adaptation of *Ustilaginoidea virens* in occupying host florets revealed by comparative and functional genomics. Nat. Commun..

[46] Cools H.J., Hammond-Kosack K.E. (2013). Exploitation of genomics in fungicide research: current status and future perspectives. Mol. Plant Pathol..

[47] Rutherford K.M., Harris M.A., Lock A., Oliver S.G., Wood V. (2014). Canto: an online tool for community literature curation. Bioinformatics.

[48] Wood V., Harris M.A., McDowall M.D., Rutherford K., Vaughan B.W., Staines D.M., Aslett M., Lock A., Bahler J., Kersey P.J. (2012). PomBase: a comprehensive online resource for fission yeast. Nucleic Acids Res..

[49] Smith B., Ashburner M., Rosse C., Bard J., Bug W., Ceusters W., Goldberg L.J., Eilbeck K., Ireland A., Mungall C.J. (2007). The OBO Foundry: coordinated evolution of ontologies to support biomedical data integration. Nat. Biotechnol..

[50] Kasprzyk A. (2011). BioMart: driving a paradigm change in biological data management. Database.

[51] Jones J.D., Dangl J.L. (2006). The plant immune system. Nature.

[52] Weßling R., Epple P., Altmann S., He Y., Yang L., Henz Stefan R., McDonald N., Wiley K., Bader Kai C. (2014). Convergent targeting of a common host protein-network by pathogen effectors from three kingdoms of life. Cell Host Microb..

